# Exercise during pregnancy mitigates the adverse effects of maternal obesity on adult male offspring vascular function and alters one‐carbon metabolism

**DOI:** 10.14814/phy2.14582

**Published:** 2020-09-25

**Authors:** Nicha P. Boonpattrawong, Saeid Golbidi, Daven C. Tai, Rika E. Aleliunas, Pascal Bernatchez, Joshua W. Miller, Ismail Laher, Angela M. Devlin

**Affiliations:** ^1^ Department of Pathology and Laboratory Medicine The University of British Columbia, and BC Children’s Hospital Research Institute Vancouver BC Canada; ^2^ Department of Family Practice The University of British Columbia, and BC Children’s Hospital Research Institute Vancouver BC Canada; ^3^ Department of Pediatrics The University of British Columbia, and BC Children’s Hospital Research Institute Vancouver BC Canada; ^4^ Department of Anesthesiology, Pharmacology and Therapeutics The University of British Columbia Vancouver BC Canada; ^5^ Department of Nutritional Sciences Rutgers University The State University of New Jersey New Brunswick NJ USA

**Keywords:** endothelial function, exercise, maternal obesity, offspring, one‐carbon metabolism

## Abstract

Maternal obesity during pregnancy can adversely affect adult offspring vascular endothelial function. This study examined whether maternal exercise during pregnancy and lactation mitigates the adverse effects of maternal obesity on offspring vascular endothelial function. Female (C57BL/6N) mice were fed from weaning a control diet (10% kcal fat) or western diet (45% kcal fat) to induce excess adiposity (maternal obesity). After 13 weeks, the female mice were bred and maintained on the diets, with and without access to a running wheel (exercise), throughout breeding, pregnancy, and lactation. Offspring were weaned onto the control or western diet and fed for 13 weeks; male offspring were studied. Maternal exercise prevented the adverse effects of maternal obesity on offspring vascular endothelial function. However, this was dependent on offspring diet and the positive effect of maternal exercise was only observed in offspring fed the western diet. This was accompanied by alterations in aorta and liver one‐carbon metabolism, suggesting a role for these pathways in the improved endothelial function observed in the offspring. Obesity and exercise had no effect on endothelial function in the dams but did affect aorta and liver one‐carbon metabolism, suggesting the phenotype observed in the offspring may be due to obesity and exercise‐induced changes in one‐carbon metabolism in the dams. Our findings demonstrate that maternal exercise prevented vascular dysfunction in male offspring from obese dams and is associated with alterations in one‐carbon metabolism.

## Graphical abstract

This study examined whether maternal exercise during pregnancy and lactation mitigates the adverse effects of maternal obesity on offspring vascular endothelial function. Maternal exercise prevented the adverse effects of maternal obesity on adult male offspring vascular endothelial function. This was accompanied by alterations in aorta and liver one‐carbon metabolism, suggesting a role for these pathways in the improved endothelial function observed in the offspring

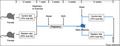

## 1 INTRODUCTION

Maternal obesity (BMI ≥ 30 kg/m^2^) at the time of conception and during pregnancy may adversely affect the cardiovascular health of women and has a long‐term impact on the health of the child (Fleming et al., 2018). For example, a large Scottish epidemiological study (*n* = 13,718) reported increased risk of hospitalization from cardiovascular events (angina, myocardial infarction, stroke or cerebrovascular disease, peripheral artery disease, and other cardiovascular diseases) and greater all‐cause mortality in adult men and women from mothers with obesity at the first antenatal visit (Reynolds et al., 2013). Similarly, the Jerusalem Perinatal Family Follow‐up study reported higher systolic and diastolic blood pressures in adult male and female children (*n* = 1,256) from women with prepregnancy obesity (Hochner et al., 2012).

Endothelial dysfunction enhances platelet aggregation, proinflammatory responses, and impairs endothelium‐dependent vasodilatation (Godo & Shimokawa, 2017). In humans, vascular endothelial function is often assessed by brachial artery flow‐mediated dilatation and impairment is a very early indicator of vascular damage and atherosclerosis (Daiber et al., 2017; Widlansky, Gokce, Keaney, & Vita, 2003). Vascular endothelial cells regulate vascular tone through the release of both vasodilators and vasoconstrictors, which in turn modulate the contractive state of smooth muscle cells (Bonetti, Lerman, & Lerman, 2003; Davignon & Ganz, 2004). Endothelial cells produce nitric oxide (NO), a major vasodilator, from L‐arginine through the action of endothelial nitric oxide synthase (eNOS) and the cofactor, tetrahydrobiopterin (BH_4_). Dihydrobiopterin is produced during the production of NO and is recycled back to BH_4_ by dihydrofolate reductase (DHFR). If this pathway is disrupted, eNOS may generate superoxide instead of NO through a process called “eNOS uncoupling” (Vásquez‐Vivar et al., 1998) and lead to impaired endothelial‐dependent vasodilatation. Dihydrofolate reductase is also required for the metabolism of folates and functions to convert dihydrofolate (DHF) to tetrahydrofolate (THF). Folate metabolism is linked to the methionine cycle, and together these metabolic pathways are often referred to as one‐carbon metabolism. These pathways are required for nucleic acid synthesis, amino acid balance, and S‐adenosylmethionine (AdoMet) synthesis (Ducker & Rabinowitz, 2017).

Studies in animal models demonstrate impaired vascular endothelial function in adult male and female offspring from mothers fed a high‐fat diet before and during pregnancy (45%–60% energy from fat), a model of of maternal gestational obesity (Fan et al., 2013; Khan, Dekou, Hanson, Poston, & Taylor, 2004; Samuelsson et al., 2008; Taylor, Khan, Hanson, & Poston, 2004; Torrens et al., 2012). For example, adult offspring from female Sprague‐Dawley rats (Taylor et al., 2004) or C57BL/6J mice (Samuelsson et al., 2008) with diet‐induced obesity have impaired endothelial‐dependent vasodilatation of mesenteric arteries, compared to offspring from dams fed a low fat chow diet. Furthermore, the impact of maternal obesity on offspring vascular function may also be influenced by offspring diet. Nonhuman primate offspring, fed a high‐fat diet from weaning until 13 months of age, that were from mothers with obesity had impaired endothelial‐dependent relaxation of abdominal aorta compared to offspring fed a low fat control diet that were also from mothers with obesity (Fan et al., 2013).

Exercise is accepted as a nonpharmacological means of improving vascular endothelial function and reducing atherosclerosis (Guizoni et al., 2016; Laufs et al., 2004; Napoli et al., 2004; Padilla et al., 2016). For example, forced exercise (swimming) reduced aortic lesion area (Napoli et al., 2004) and treadmill training improved thoracic aorta endothelial‐dependent vasodilatation (Guizoni et al., 2016) in low density lipoprotein receptor‐deficient mice. Futhermore, female C57BL/6 mice fed a western diet with access to a running wheel (voluntary exercise) had lower endothelial cortical stiffness in thoracic aorta explants and lower femoral artery stiffness compared to sedentary mice (Padilla et al., 2016).

Variable findings have been reported from the few studies investigating the effects of maternal exercise during pregnancy on offspring vascular function (Bahls et al., 2014; Blaize et al., 2015). Adult male and female swine offspring from sows fed a standard chow diet that completed aerobic exercise treadmill training during pregnancy had lower endothelial‐independent vasodilatation, but no differences in endothelial‐dependent vasodiliation, of femoral arteries compared to offspring from sedentary sows were observed (Bahls et al., 2014). In contrast, no differences in vascular endothelial‐dependent or independent vasodilatation of the abdominal aorta were observed between adult male and female Sprague‐Dawley rat offspring from sedentary dams and those from dams that exercised voluntarily (Blaize et al., 2015).

Exercise may be a useful means to improve vascular dysfunction in offspring from mothers with obesity. However, little is known about the impact of exercise during pregnancies complicated with obesity. In this study, we test the hypothesis that maternal exercise mitigates the adverse effects of maternal obesity on offspring vascular endothelial function and alters one‐carbon metabolism.

## 2 MATERIALS AND METHODS

### 2.1 Mice and diets

**FIGURE 1:**
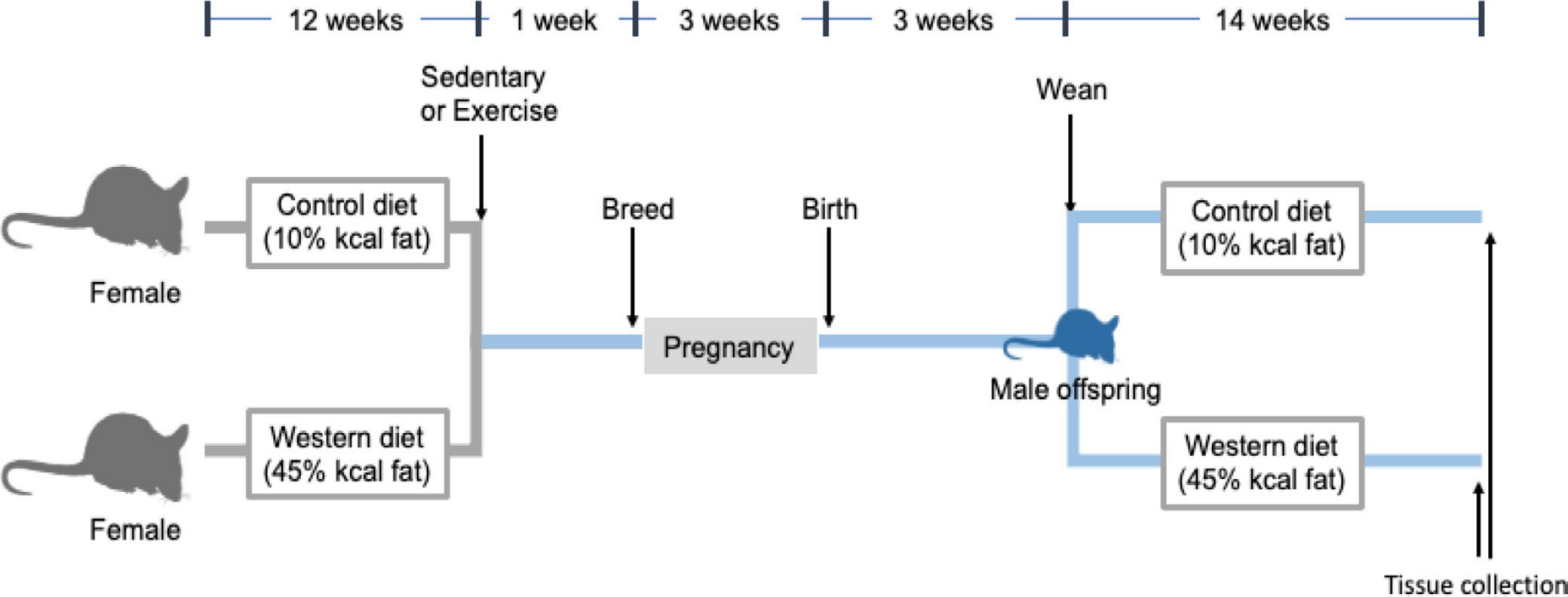
Study design. Female C57BL/6N mice (dams) were fed either a control (control dams) or western diet(obese dams) from weaning (age 3 weeks). At 12‐weeks post weaning, dams were put into cages with (exercise) or without (sedentary) a running wheel. The dams were bred with male mice fed the control diet 1 week later. Dams had access to the running wheel throughout breeding, pregnancy and lactation. Offspring were weaned at age 3 weeks onto the control diet or western diet and fed for 14 weeks

Female C57BL/6N mice were fed from weaning a control diet (10% kcal fat, D12450K Research Diets) or a western diet (45% kcal fat, D12451 Research Diets). After 13 weeks on the diet, female mice were bred with age‐matched male mice fed the control diet. A week before breeding, female mice were housed individually, with or without access to a running wheel for voluntary exercise throughout breeding, pregnancy, and lactation. A schematic representation of the study design is presented in Figure 1. The distance and time on the running wheel were tracked with an odometer (YT‐816, BoGeer) attached to the wheel. There was no effect of diet or exercise on litter size and sex distribution of the offspring. Offspring mice were weaned (aged 3 weeks) and fed either the control or western diet for 13–15 weeks. One male offspring from each litter was analyzed. Mice were housed under a standard 12‐hr light, 12‐hr dark cycle and had ad libitum access to food and water. Dams and offspring were weighed weekly. At the end of the feeding period, mice were anesthetized with isofluorane and nonfasting blood was collected via cardiac puncture. Blood was left at room temperature to coagulate for 15 min, then centrifuged at 10,000 *g* at 4°C for 10 min to obtain the serum. Tissues were flash‐frozen in liquid nitrogen and stored at −80°C until further analyses. All procedures were performed according to the guidelines and with the approval of the University of British Columbia Animal Care Committee.

### 2.2 Body composition and glucose tolerance in the dams

Body composition was quantified by quantitative magnetic resonance imaging (EchoMRI‐100 Echo Medical Systems) and intraperitoneal glucose tolerance tests were conducted (Henderson et al., 2018) in the dams at age 23–25 weeks (after the pups were weaned). Briefly, for the intraperitoneal glucose tolerance test, following a 5 hr fast, the dams were given an intraperitoneal injection of 25% dextrose solution at a dose of 1 g/kg lean mass and blood glucose was measured via tail puncture with a glucometer (Bayer) at baseline (0 min), 15, 30, 60, 90, and 120 min post injection.

### 2.3 Biochemical assays

Citrate synthase activity was quantified in quadriceps muscles from the dams using MitoCheck Citrate Synthase Activity Assay Kit (Cayman Chemical). Serum nitrate/nitrite concentrations were quantified by commercial colorimetric assay (Cayman Chemical). Serum total homocysteine and cysteine were quantified by high pressure liquid chromatography with postcolumn fluorescence detection as previously described (Gilfix, Blank, & Rosenblatt, 1997).

### 2.4 Vascular function

Vascular function was assessed in rings of thoracic aorta by isometric force measurements using a wire myograph (Sallam, Fisher, Golbidi, & Laher, 2011). Briefly, aortic rings were preconstricted with phenylephrine (10^–5^ M) to reach maximal contraction followed by addition of acetylcholine (ACh, 10^–10^–10^–5^ M) or sodium nitroprusside (SNP, 10^–10^–10^–5^ M) to assess endothelium‐dependent and endothelium‐independent vasodilatation, respectively. Vasoconstriction was assessed by comparing vasoconstriction in response to phenylephrine (10^–10^–10^–5^ M) to maximal vasoconstriction in response to KCl (80mM). To assess the contribution of endogenous nitric oxide, aortic rings were also incubated with the NOS inhibitor, N^G^‐nitro‐_L_‐arginine methyl ester (L‐NAME), then constricted with increasing concentration of phenylephrine (10^–10^–10^–5^ M).

### 2.5 Endothelial cell isolation

Aorta (arch, thoracic and abdominal portions) were harvested, adipose tissue and connective tissue removed, followed by digestion with aortic dissociation enzyme solution (hyaluronidase, DNase I, and Liberase in PBS, Sigma; Butcher, Herre, Ley, & Galkina, 2011) for 15 min at 37°C to obtain a suspension of single cells. Endothelial cells were purified by fluorescence activated cell sorting (FACS) using a FACSAria flow cytometer (BD Biosciences). Cells were incubated with anti‐mouse CD16/CD32 (eBiosciences) to block Fc receptors and incubated with the following antibodies on ice for 2 hr: anti‐CD31‐APC (clone 390, eBioscience), anti‐CD105‐PE (clone MJ7/18, eBiosciences), and anti‐CD45‐FITC (clone 30‐F11, eBiosciences) prior to sorting. Dead cells were excluded by staining cells with 7AAD (ebioscience). Cells were directly sorted into TRIzol reagent (Thermofisher) for extraction of RNA. Sorted endothelial cell functionality was confirmed by acetylated‐low density lipoprotein (Ac‐LDL) uptake assay (Kovacs‐Kàsa, Varn, Verin, & Gonzales, 2017; Voyta, Via, Butterfield, & Zetter, 1984) in separate experiments. Briefly, fluorescently labeled Ac‐LDL (Thermofisher) was added to the endothelial cell suspension at a final concentration of 10ug/ml and incubated for 2 hr in an atmosphere of 5% CO_2_ at 37°C. LDL uptake by the cells was then analyzed using flow cytometry. Human umbilical vein endothelial cells (HUVEC) were used as a positive control.

### 2.6 Nanostring gene expression analysis

Total RNA was extracted from purified endothelial cells using TRIzol reagent and RNeasy Micro kit (Qiagen). A custom‐designed Nanostring CodeSet was used to quantify mRNA expression of 95 genes involved in vascular endothelial function, oxidative stress, inflammation, and epigenetic regulation (Table S1). Due to the low number of cells isolated from a single mouse aorta, total RNA from endothelial cells was converted to cDNA and multiplex target enrichment was performed with custom‐designed primers from NanoString to amplify target genes in the panel by eight rounds of amplification using the TaqMan PreAmp MasterMix (Applied Biosystems^TM^; Porras, Kaur, Ring, Schechter, & Lang, 2018). The amplified endothelial cell cDNA was hybridized to probes at 65°C overnight and analyzed using the nCounter SPRINT Profiler (NanoString). Quality control assessment and data normalization were performed using nSolver Analysis Software (NanoString Technologies). The raw NanoString counts were first checked for quality control. The counts for a target gene must be higher than 2 standard deviations above the spiked in negative controls for the software to detect gene expression. The counts were then normalized to two housekeeping genes (*Actb* and *Gapdh*) and log2 transformed.

### 2.7 Quantification of gene expression

Real‐time PCR and the ^ΔΔ^Ct method of relative quantification were used to quantify mRNA levels of target genes (Livak & Schmittgen, 2001; Schmittgen & Livak, 2008). Total RNA was extracted from liver and whole mouse aorta and using RNeasy Mini Kit (Qiagen). Total RNA (1,000 ng) was reverse transcribed using a high‐capacity cDNA reverse transcription kit (Applied Biosystems). TaqMan Gene Expression Master Mix (Applied Biosystems) and the following mouse‐specific primers (Integrated DNA Technologies) were used to quantify mRNA levels in the liver: *Cbs* (Mm.PT.58.5852051), *Mat1a* (Mm.PT.58.10231439), *Mthfr* (Mm.PR.58.16943023), *Mtrr* (Mm.PT.58.31931134), with 18S rRNA (Mm04277571; Thermofisher) as an endogenous control. Samples from each mouse were run in duplicate; gene expression analyses were repeated twice for each mouse, and the means of the two experiments used. The same protocol was followed to quantifiy mRNA levels in aorta with the following primers (Integrated DNA Technologies): *Mthfd1* (Mm.PT.58.8705049) and *Dhfr* (Mm.PT.58.41807392) with *Actb* (Mm.PT.39a.2221483) as an endogenous control.

### 2.8 Statistical analyses

Offspring fed the control diet were analyzed separately from offspring that were fed the western diet. The effects of maternal diet and maternal exercise on offspring outcomes were assessed by two‐way analysis of variance (ANOVA). If no interaction between the variables were observed, the *p*‐values for the main effects of maternal diet and maternal exercise are stated. If a significant interaction between maternal diet and maternal exericise was found, the *p*‐value for the interaction is provided. The effect of maternal exercise was determined separately in offspring mice from dams fed the control diet and offspring mice from dams fed the western diet by *t* test and *p*‐values are provided if the models were significant. Repeated measures two‐way ANOVA was used to analyze vascular function data and glucose tolerance data with maternal diet and maternal exercise as the independent variables. For all models, a value of *p* < .05 was considered statistically significant. Statistical analyses were performed using SPSS software (IBM). Individual data points are presented and the bar graphs represent the mean ± *SD*.

## 3 RESULTS

### 3.1 Exercise lowers adiposity but has no effect on glucose tolerance or vascular function in the dams

**FIGURE 2:**
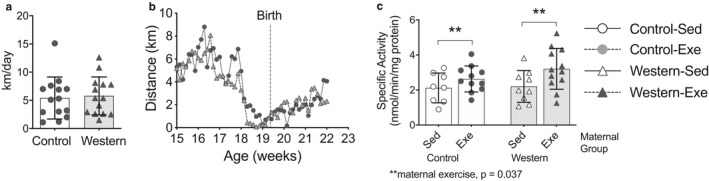
Exercise during pregnancy and lactation in the dams. (a) Average running distance per day during the first week of exercise and (b) average distance ran per day from one week before breeding until the end of lactation. (c), Citrate synthase activity in quadricep muscle. Values for each individual mouse presented; bar graphs represent mean ± *SD* (*n* = 8–14/group)

Dams were acclimatized to running wheels one week prior to breeding at age 16 weeks. Diet had no effect on the daily distances the dams ran throughout breeding, pregnancy, and lactation (Figure 2a,b). Greater citrate synthase activity, a common marker of mitochondrial content (Tanner et al., 2013) that confirms exercise, was observed in quadriceps muscle from dams that exercised compared to sedentary dams (*p* = .037; Figure 2c).

**FIGURE 3:**
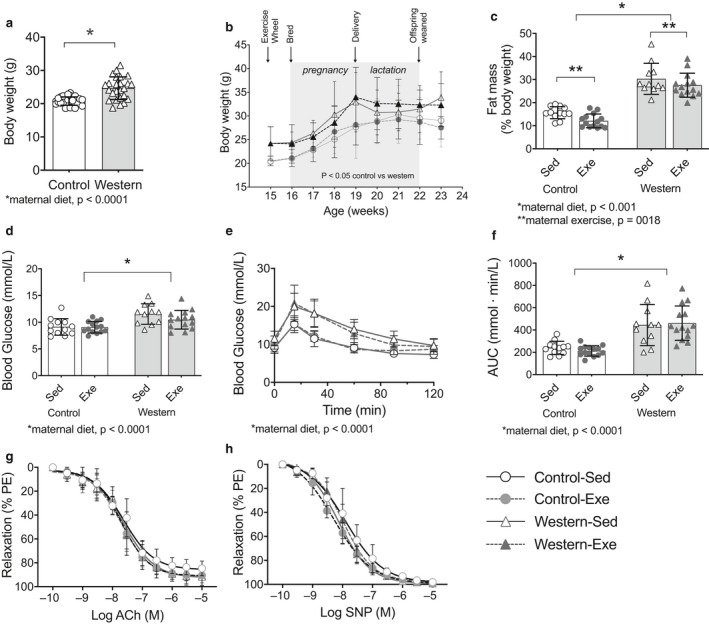
Exercise during pregnancy and lactation lowered fat mass but had no effect on glucose tolerance or thoracic aorta endothelial‐dependent and independent vasodilatation in the dams. (a) Body weight at 15 weeks of age, just prior to exercise; and (b) throughout breeding, pregnancy and lactation. (c) Total body fat percentage at the end of lactation. (d) Fasting blood glucose; (e) intraperitoneal glucose tolerance test; and (f) area under the glucose excursion curve at the end of lactation. (g) Acetylcholine (Ach)‐induced and (h) sodium nitroprusside (SNP)‐induced vasodilatation of aortic rings preconstricted with phenylephrine (10^–5^ M) at age 23 weeks. Values for each individual mouse presented; bar graphs represent mean ± *SD* (*n* = 8–14/group). For vascular function graphs, data symbols represent mean ± *SD*. Exe, exercise; Sed, sedentary

At age 15 weeks, just prior to the exercise intervention, dams fed the western diet had significantly higher body weight (24.7 ± 3.4 vs. 20.8 ± 1.3 g, *p* < .0001) compared to those fed the control diet (Figure 3a). The diet‐induced differences in body weight were maintained thoughout pregnancy and lactation (Figure 3b); confirming our model of maternal diet‐induced obesity. Body composition was assessed in the dams after the pups were weaned at the end of lactation. As expected, sedentary dams fed the western diet had a higher percent fat mass compared to dams fed the control diet (*p* < .0001). Exercise lowered the percent fat mass in both western and control diet‐fed dams (*p* = .018); Figure 3c).

We also assessed glucose tolerance in the dams when the pups were weaned. Fasting blood glucose was greater (*p* < .0001) in obese dams compared to control dams (Figure 3d). The obese dams also had greater glucose intolerance compared to control dams, as shown in the glucose excursion curve (*p* < .001) and AUC (*p* < .0001; Figure 3e,f). No effect of exercise was observed.

Vascular endothelial‐dependent and endothelial‐independent dilatation were assessed in aortic rings from dams in response to increasing concentrations of acetylcholine and sodium nitroprusside, respectively. No effect of diet or exercise were observed (Figure 3g,h).

### 3.2 Maternal exercise improves offspring vascular endothelial function in offspring fed the western diet

**FIGURE 4:**
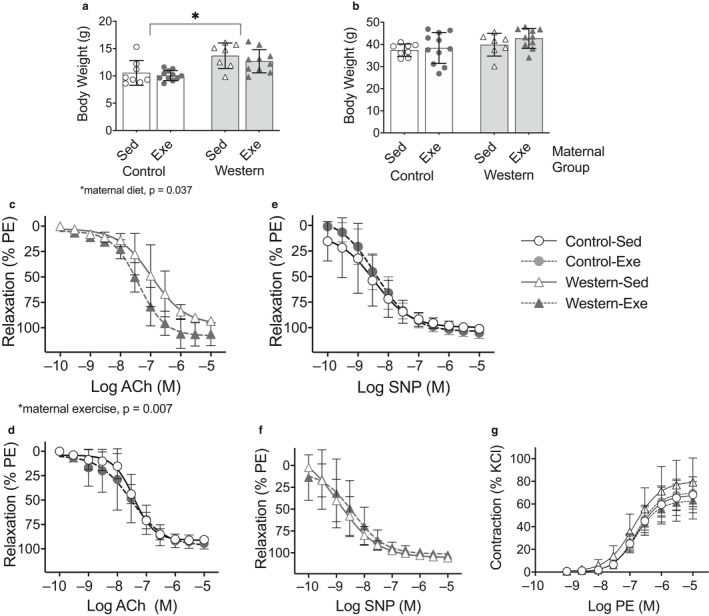
Exercise in dams with diet‐induced obesity improved thoracic aorta endothelial‐dependent vasodilatation in adult male offspring fed the western diet. (a) Offspring body weight at weaning; and (b) in adulthood (age 17 weeks). Offspring were fed the western diet. Endothelial‐dependent vasodilatation in response to increasing acetylcholine (Ach) concentrations in (c) offspring from dams with diet‐induced obesity and (d) offspring from control dams . Endothelialindependent vasodilatation in response to increasing sodium nitroprusside (SNP) concentrations in (e) offspring from dams with diet‐induced obesity and (f) offspring from control dams. (g) Vasoconstriction assessed in response to phenylephrine. Bar graphs represent mean ± *SD*, values for each individual mouse presented; (*n* = 7–11/group). For wire myograph curves, symbols represent mean ± *SD* (*n* = 5–6/group) at each drug concentration

**FIGURE 5:**
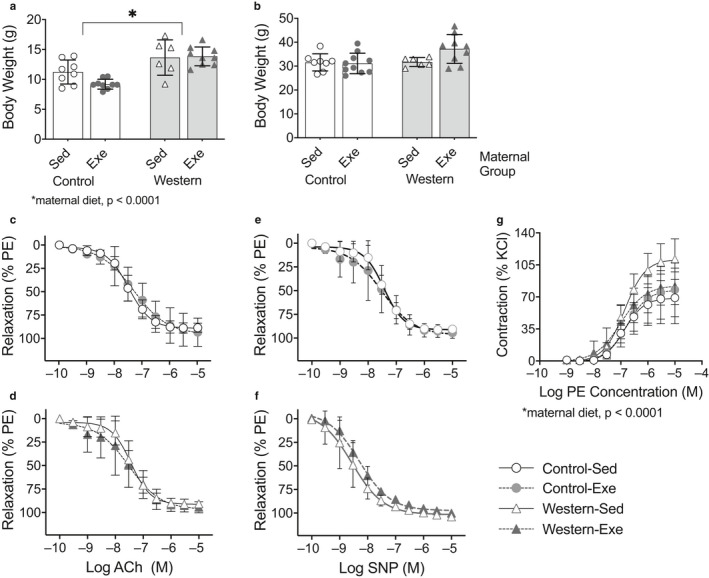
Exercise in dams with diet‐induced obesity had no effect on thoracic aorta endothelial‐dependent vasodilatation in adult male offspring fed the control diet. (a) Control diet‐fed offspring body weight at weaning and (b) at age 14 weeks. At age 14–15 weeks, aortic rings were harvested from offspring and vasodilatation were assessed in response to (c,d) acetylcholine (Ach) and (e,f) sodium nitroprusside (SNP) for endothelial‐dependent and endothelial‐independent vasodilatation, respectively. (g) Vasoconstriction assessed in response to phenylephrine . For bar graphs, values for each individual mouse presented; bar graphs represent mean ± *SD* (*n* = 7–11/group). Symbols represent mean ± *SD* (*n* = 5–6/group) at each drug concentration. Data were analyzed by repeated measures ANOVA and symbols represent mean ± *SD*

Greater body weight at weaning was observed in offspring from obese dams compared to those from control dams that were weaned onto the western diet (*p* = .037; Figure 4a) or control diet (*p* < .0001; Figure 5a); there was no effect of maternal exercise (Figures 4a and 5a). There was no effect of maternal diet or exercise on body weight in adult offspring fed the western diet (Figure 4b) or control diet (Figure 5b).

We observed an interaction (*p* = .007) between maternal diet and exercise on endothelial‐dependent vasodilatation in offspring fed the western diet in the 2‐way ANOVA model. Therefore, the effect of exercise was assessed separately in offspring from obese and control dams. Offspring from obese dams that exercised had greater endothelial‐dependent vasodilatation compared to offspring from obese sedentary dams (*p* = .007; Figure 4c). There was no effect of maternal diet or exercise on SNP‐induced vasodilatation in the offspring (Figure 4e), suggesting the effect of maternal exercise on vascular function was specific to the endothelium. In contrast, maternal exercise had no effect on endothelial‐dependent or endothelial‐independent vasodilatation in offspring from control dams (Figure 4d,f). There was no effect of maternal diet‐induced obesity or exercise on vasoconstriction in response to phenylephrine (PE) (Figure 4g).

In offspring fed the control diet, there was no effect of maternal diet or exercise on endothelial‐dependent or endothelial‐independent vasodilatation (Figure 5c–f). However, greater vasoconstriction in response to PE was observed in offspring from obese dams compared to offspring from control dams (*p* < .0001); no effect of maternal exercise was observed (Figure 5g). These findings suggest that the effect of maternal exercise during gestation on offspring vascular function is dependent on the offspring's diet.

### 3.3 Maternal exercise increases offspring NO levels

**FIGURE 6:**
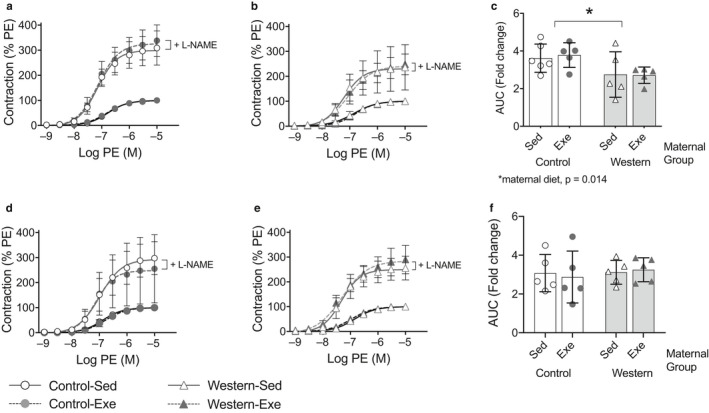
Maternal diet‐induced obesity reduced basal nitric oxide production in thoracic aorta from adult male offspring fed the western diet. Phenylephrine (PE)‐induced contraction in aortic rings, with or without the nitric oxide synthase inhibitor NG‐nitro‐Larginine methyl ester (L‐NAME), from (a,b) offspring fed the western diet or (d,e) control diet. Difference in the area under the curve (AUC) between contraction curves with and without L‐NAME in offspring fed the (c) western diet or (f) control diet. For contraction curves, symbols represent mean ± *SD* for each group at each PE concentration. For bar graphs, values for each mouse presented; bar graphs represent mean ± *SD* (*n* = 5–6/group)

**FIGURE 7:**
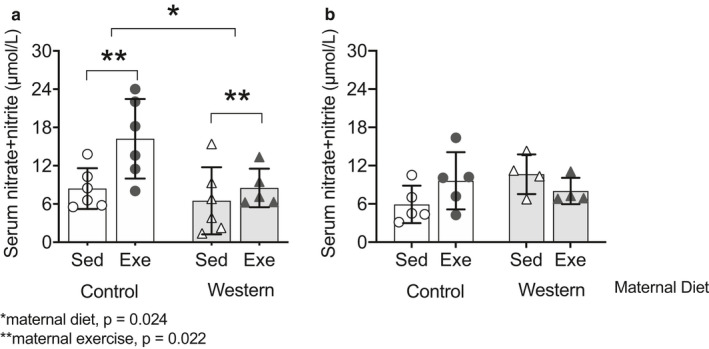
Maternal exercise increased serum nitrate and nitrite concentrations in male offspring fed (a) the western diet but not in those fed (b) the control diet. Values for each mouse presented; bar graphs represent mean ± *SD* (*n* = 4–6/group)

We determined if maternal exercise improved offspring endothelial function through effects on basal NO production using aortic rings. We assessed PE‐induced vasoconstriction before and after incubation of aortic rings with L‐NAME, and calculated the difference between the area under each curve (AUC) as an indication of basal NO production. In offspring fed the western diet, those from obese dams had a smaller difference in contraction between the phenylephrine curves, with and without L‐NAME, compared to offspring from control dams (*p* = .014; Figure 6a–c); we observed no effect of maternal exercise. This suggests that the improved vascular endothelial function observed in offspring from obese dams that exercised does not involve changes in basal NO production in the aorta. We further assessed serum nitrate and nitrite concentrations as an indicator of circulating total NO concentrations. In offspring fed the western diet, serum nitrate and nitrite concentrations were lower in those from obese dams compared to control dams (*p* = .024; Figure 7a). Further, offspring from obese and control dams that exercised had higher serum nitrate and nitrite concentrations than offspring from sedentary dams (*p* = .022; Figure 7a). This suggests some effects of maternal exercise on offspring NO production.

In contrast, there were no effects of maternal exercise or diet on PE‐induced vasoconstriction, with and without L‐NAME in offspring fed the control diet (Figure 6d–f). We also observed no effect of maternal obesity or exercise on serum nitrate and nitrite concentrations in offspring fed the control diet (Figure 7b).

### 3.4 Maternal exercise alters endothelial cell gene expression patterns in adult offspring fed the western diet

**FIGURE 8:**
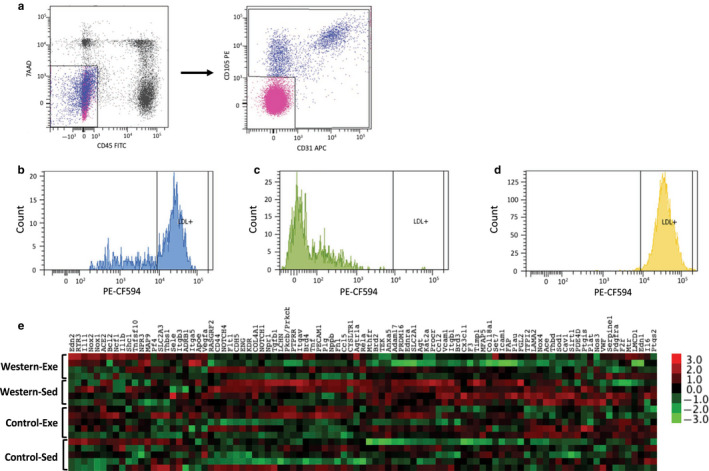
Aortic endothelial cell gene expression profile in offspring fed the western diet. (a) Representative scattergram of FACS analyses to isolate aortic endothelial cells. Cells were first gated to exclude CD45+ and 7AAD+ cells then CD31+, CD105+, and CD31+/CD105+ were sorted as endothelial cell population. Isolated endothelial cell functionality was confirmed by ac‐dil‐LDL uptake assay in sorted (b) endothelial cells and (c) CD45‐/CD31‐/CD105‐. (d) Gene expression analysis of isolated aortic endothelial cells using a NanoString panel and normalized to housekeeping genes

To further understand how exercise in obese dams improved vascular endothelial function in the adult offspring fed the western diet, we assessed gene expression patterns in aortic endothelial cells. The extracellular markers CD31 (PECAM1) and CD105 (endoglin) were used to identify aortic endothelial cells (Marelli‐Berg, Peek, Lidington, Stauss, & Lechler, 2000). Leukocytes and dead cells were excluded using CD45 and 7AAD, respectively. Figure 8a shows a representative sequence of FACS sorting. We selected for combined populations of CD31^+^/CD105^−^, CD31^−^/CD105^+^, and CD31^+^/CD105^+^ cells as the endothelial cell population. Functionality of isolated endothelial cells was confirmed by labeled Dil‐Ac‐LDL uptake using flow cytometry (Okaji et al., 2004). Sorted endothelial cells show a higher population of cells positive for Dil‐Ac‐LDL compared to CD45^−^/CD31^−^/CD105^‐^ population (70.9% vs. 0%; Figure 8b,c). Cultured HUVEC were used as positive control and to determine the gating area for Dil‐Ac‐LDL^+^ cells (Figure 8d). These results confirmed our endothelial cell population.

We assessed gene expression patterns in isolated endothelial cells by digital PCR using the NanoString nCounter system (Figure 8e). The effect of maternal exercise was determined separately in offspring from obese dams and control dams. Of the 95 genes that were assessed, 13 genes were differentially expressed in offspring from obese dams that exercised compared to sedentary obese dams. Specifically, offspring from obese dams that exercised had lower expression of genes involved in vasoconstriction (*Ace*), angiogenesis (*Itgb1*, *Mfap5*), oxidative stress (*Nox4*, *Sod1*), coagulation (*Anxa5*, *Tfpi2*, *Thbd*, *Timp1*), inflammation (*Ccl2*, *Tgfb1*, *Vcam1*) and one‐carbon metabolism (*Mthfr*) compared to offspring from sedentary obese dams (Figure 8e; Table 1). In contrast, minimal differences in gene expression were observed in offspring from control dams; only two genes were differentially expressed in offspring from dams that exercised compared to sedentary dams. Lower *Ccl2* expression and higher *Apoe* expression were observed in offspring from control dams that exercised compared to offspring from sedentary control dams (Figure 8e; Table 1). There was no effect of maternal diet or exercise on *Nos3* expression (encodes eNOS; Table 1). These findings demonstrate that offspring from dams that exercised during pregnancy/lactation have a unique endothelial cell gene expression profile.

**TABLE 1:** Expression of genes in isolated endothelial cells from mice with diet‐induced obesity

Gene	Maternal control diet		Maternal western diet	
	Sedentary	Exercise	Sedentary	Exercise
*Ace*	11.77 ± 0.08	12.24 ± 0.73	13.13 ± 0.36	11.10 ± 0.54**
*Anxa5*	9.11 ± 0.30	8.96 ± 1.15	9.99 ± 0.53	7.55 ± 0.71**
*Apoe*	8.56 ± 0.35	10.00 ± 0.33**	9.42 ± 0.57	10.12 ± 0.94
*Ccl2*	5.76 ± 0.64	2.62 ± 0.61**	6.85 ± 0.47	3.34 ± 0.81**
*Itgb1*	12.94 ± 0.84	13.02 ± 0.45	14.09 ± 0.27	12.31 ± 0.62**
*Mfap5*	8.38 ± 0.50	8.21 ± 0.68	8.85 ± 0.27	7.38 ± 0.53**
*Mthfr*	6.73 ± 0.86	7.19 ± 0.60	8.21 ± 0.44	6.13 ± 0.58**
*Nos3*	6.99 ± 0.61	7.60 ± 0.34	7.60 ± 0.55	6.98 ± 0.84
*Nox4*	8.57 ± 0.62	9.09 ± 1.13	9.77 ± 0.52	7.66 ± 0.77**
*Sod1*	10.89 ± 0.53	11.19 ± 0.41	11.65 ± 0.08	10.39 ± 0.24**
*Tfpi2*	3.34 ± 1.53	3.65 ± 1.88	5.81 ± 0.83	3.18 ± 0.56**
*Tgfb1*	10.74 ± 1.42	10.84 ± 0.99	11.80 ± 0.70	10.24 ± 0.39**
*Thbd*	9.96 ± 0.58	10.13 ± 1.02	10.81 ± 0.40	9.33 ± 0.24**
*Timp1*	8.30 ± 0.65	8.35 ± 0.95	9.59 ± 0.83	7.65 ± 0.19**
*Vcam1*	6.02 ± 0.48	6.02 ± 0.76	7.00 ± 0.18	5.70 ± 0.29**
*Vwf*	10.01 ± 1.12	10.21 ± 0.40	10.08 ± 1.22	9.98 ± 0.57

Note

Values are log2 counts, geometric mean ± *SD* (*n* = 4–5 mice per group).

** Main effect of maternal exercise, *p* < .05.

### 3.5 Maternal exercise affects one‐carbon metabolism in adult offspring fed the western diet

**FIGURE 9:**
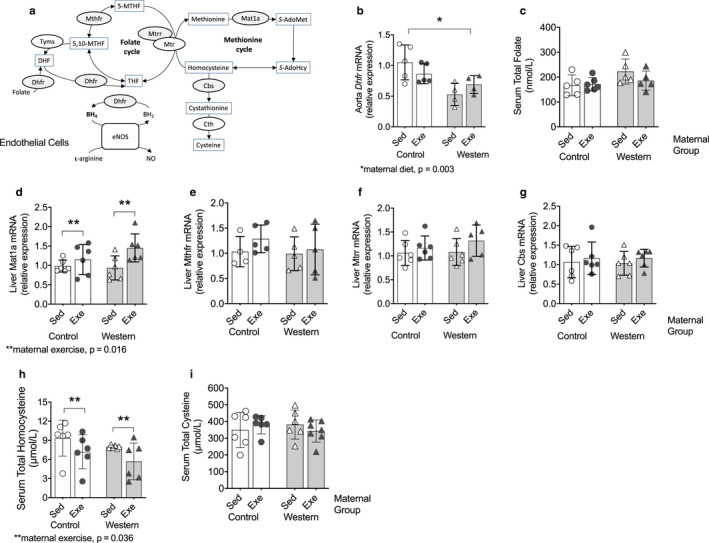
Maternal exercise alters one‐carbon metabolism in adult offspring fed the western diet. (a) Schematic simplified representation of one‐carbon metabolism. (b) Aorta Dhfr mRNA. (c) Serum total folate concentrations. Liver (d) Mat1a, (e) *Mthfr*, (f) *Mtrr*, and (g) *Cbs* mRNA. Serum total (h) homocysteine and total (i) cysteine concentrations. Values for each individual mouse presented; bar graphs represent mean ± *SD* (*n* = 4‐6/group). Abbreviations: BH_2_, dihydrobiopterin; BH_4_, tetrahydrobiopterin; Cbs, cystathionine β‐synthase; Cth, cystathionine γ‐lyase; Dhfr, dihydrofolate reductase; eNOS, endothelial nitric oxide synthase; Mat1a, methionine adenosyltransferase; 5‐MTHF, 5‐methyltetrahydrofolate; 5,10‐MTHF, 5,10‐methylenetetrahydrofolate; Mthfr, methylenetetrahydrofolate reductase; Mtr, methionine synthase; Mtrr, methionine synthase reductase; NO, nitric oxide; *S*‐AdoHcy, S‐adenosylhomocysteine; *S*‐AdoMet, Sadenosylmethionine; THF, tetrahydrofolate

Through the requirement of DHFR for the production of BH_4_, endothelial NO synthesis is linked to folate and one‐carbon metabolism (Figure 9a). We found lower *Dhfr* mRNA in offspring from obese dams than offspring from control dams; no effect of maternal exercise was observed (*p* = .003; Figure 9b). There was no effect of maternal diet or exercise on offspring serum total folate concentrations (Figure 9c) suggesting the effects of maternal exercise and obesity are specifc for vascular folate metabolism.

We then assessed the hepatic gene expression of key enzymes required for one‐carbon metabolism because of the central role of the liver in the metabolism of one‐carbon nutrients (Ducker & Rabinowitz, 2017). We found that offspring from control and obese dams that exercised had higher hepatic *Mat1a* mRNA (*p* = .016; Figure 9d), which encodes methionine adenosyltransferase (MAT) an enzyme required for the synthesis of AdoMet. No differences in offspring liver *Mthfr* (Figure 9e), *Mtrr* (Figure 9f) or *Cbs* mRNA (Figure 9g) were observed. Lower serum total homocysteine concentrations (*p* = .36; Figure 9h) were found in offspring from control and obese dams that exercised; no effects on serum total cysteine concentrations were observed (Figure 9i).

### 3.6 Exercise during pregnancy and lactation affects one‐carbon metabolism in the dams

**FIGURE 10:**
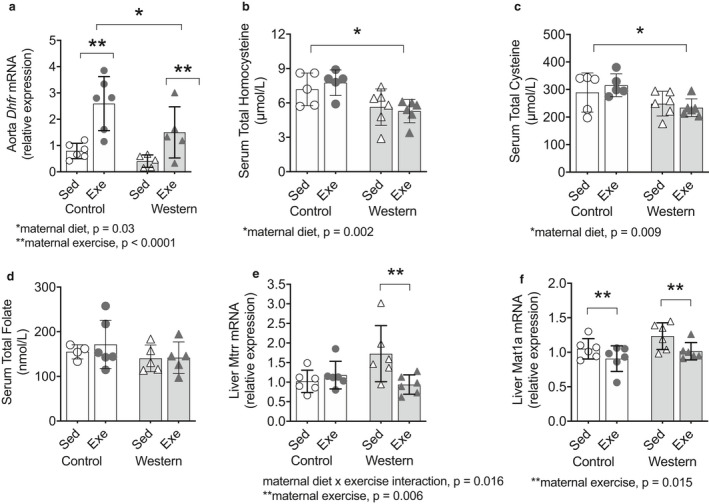
Exercise during pregnancy altered mRNA expression of one‐carbon metabolism in the dams. (a) Aortic Dhfr (dihydrofolate reductase) mRNA expression in dams. Serum total (b) homocysteine, (c) cysteine, and (c) folate concentrations in dams. Hepatic mRNA expression of (e) Mtrr (methionine synthase reductase) and (f) *Mat1a* (methionine adenosyltransferase) in dams aged 23–26 weeks. Values for each individual mouse presented; bar graphs represent mean ± *SD* (*n* = 5–6/group)

Given the effects of maternal exercise and obesity on aorta and liver one‐carbon metabolism in the offspring, we determined if these alterations occurred secondary to an effect of exercise and obesity on one‐carbon metabolism in the dams. Obese dams had lower *Dhfr* mRNA in aorta than control dams (*p* = .03; Figure 10a). Obese and control dams that exercised had higher *Dhfr* mRNA in aorta compared to sedentary obese and control dams, respectively (*p* < .0001; Figure 10a). In contrast to what we observed in the offspring, we found no effect of exercise on serum total homcoysteine concentrations in the dams (Figure 10b). However, we found lower serum total homocysteine (*p* = .002; Figure 10b) and cysteine (*p* = .009; Figure 10c) concentrations in obese dams compared to control dams. Maternal diet and exercise had no effect on serum total folate concentrations (Figure 10d).

We further assessed liver one‐carbon metabolism in the dams. We observed an interaction (*p* = .016) between the effects of exercise and diet on liver *Mtrr* mRNA in the dams. Obese dams that exercised had lower *Mtrr* mRNA (*p* = .006) compared to sedentary dams; no effect of exercise was observed in control dams (Figure 10e). Control and obese dams that exercised had lower *Mat1a* mRNA compared to sedentary dams (*p* = .015; Figure 10f).

## 4 DISCUSSION

This study examined whether maternal exercise during pregnancy and lactation mitigates the adverse effects of maternal obesity on offspring vascular endothelial function. We report three major findings in this study. First, maternal exercise mitigates the adverse effects of maternal obesity on offspring vascular endothelial function. The positive effect of maternal exercise was dependent on offspring diet and was only observed in offspring fed the western diet. Second, improvements in vascular endothelial function were associated with alterations in aorta and liver one‐carbon metabolism, suggesting a role for these pathways in the improved endothelial function observed in the offspring from exercised obese dams. Third, obesity and exercise had no effects on vascular function in the dams but did affect aorta and liver one‐carbon metabolism, suggesting the phenotype observed in the offspring may be due to obesity and exercise‐induced changes in one‐carbon metabolism in the dams.

To our knowledge, no studies have reported on whether exercise during pregnancy can mitigate the adverse effects of maternal obesity on offspring vascular endothelial function. A recent study reported that maternal treadmill training can mitigate the adverse effects of maternal obesity on cardiac function in adult male C57BL/6J mouse offspring; vascular function was not assessed (Beeson et al., 2018). Taken together with our findings, exercise during pregnancy may be an effective nonpharmacological intervention to prevent adverse cardiovascular outcomes in offspring from mothers with gestational obesity.

We developed a method to isolate primary vascular endothelial cells as a first step towards understanding the mechanisms underlying improvements in vascular endothelial function in the offspring from exercised obese dam. Surprisingly, we found no effect of maternal obesity or exercise on endothelial *Nos3* expression and basal NO production in offspring thoracic aorta. However, we did find higher serum NO concentrations, suggesting some effects of maternal exercise on NO production in the offspring. Intestingly, we also found that offspring from exercised obese dams had reduced endothelial *Sod1* mRNA (encodes superoxide dismutase 1), suggesting maternal exercise reduced oxidative stress in these offspring. Others have reported that nonpregnant mice that exercise have greater basal aorta NO levels attributed to a decrease in oxidative stress (Fukai et al., 2000; Moien‐Afshari et al., 2008). Whether exercise in the obese dams mitigated oxidative stress in vessels from the dams themselves is not known. Taken together, we postulate that exercise in obese dams improved offspring vascular function because of decreased NO degradation and reduced oxidative stress, rather than by increasing endothelial NO synthesis.

Interestingly, we identified several indications that the improvement in vascular endothelial function in the offspring from exercised obese dams involved folate and one‐carbon metabolism. We investigated *Dhfr* expression because of its role in recycling BH_4_, a required cofactor for NO synthesis. We predicted that the improvements in endothelial function in the offspring from the exercised obese dams would be accompanied by greater expression of *Dhfr* in the aorta. Overexpression of endothelial DHFR can reverse eNOS uncoupling and restore nitric oxide production (Chalupsky & Cai, 2005; Whitsett et al., 2013). Dams that exercised had greater *Dhfr* expression in the aorta than sedentary dams. However, we observed no effect of maternal exercise on aorta *Dhfr* expression in the offspring. This finding further supports our suggestion that the improvements in endothelial function in the offspring from the exercised obese dams is due to decreased NO degradation rather than effects of maternal exercise on offspring endothelial NO production.

A central function of DHFR is to catalyze the reduction of DHF to THF, which is then further metabolized by the folate cycle and used in a variety of cellular functions, including DNA synthesis and methylation reactions (Glier, Green, & Devlin, 2014). Unexpectedly, the improvements in endothelial function in offspring from the exercised obese dams were accompanied by lower endothelial *Mthfr* expression, an important enzyme in the folate cycle that functions to synthesize 5‐MTHF. The role of folate and MTHFR in endothelial function has predominantly been studied in the context of hyperhomocysteinemia (Frosst et al., 1995), which can cause endothelial dysfunction through uncoupling of eNOS (Dayal & Lentz, 2008). Whole body *Mthfr* ^+/‐^ mice fed a diet to increase plasma total homocysteine concentrations have impaired cerebrovascular endothelial function (Devlin et al., 2004). However, little is known regarding endothelial cell folate metabolism and how this influences endothelial function, independent of homocysteine. Antionaides et al. reported that endothelial function of mammary artery rings is positively associated with arterial 5‐MTHF concentrations, but not plasma 5‐MTHF or total homocysteine concentrations, and that increasing arterial 5‐MTHF concentration increases arterial BH_4_ concentration (Antoniades et al., 2009; Antoniades et al., 2006). This suggests that endothelial 5‐MTHF may play an important role in endothelial function.

Exercise has been reported to reduce circulating total homocysteine concentrations in humans and in mice (Chan et al., 2018; Neuman, Albright, & Schalinske, 2013; Okura et al., 2006). We found that offspring from dams that exercised had lower serum total homocysteine concentrations than offspring from sedentary dams.This was despite no effect of exercise on serum total homocysteine concentrations in the dams. The liver is a major source of circulating homocysteine (Mudd et al., 2000), leading us to also investigate whether there were changes in hepatic enzymes in one‐carbon metabolism. Homocysteine has two metabolic fates in the liver: remethylation to methionine, or transsulfuration and conversion to cysteine. We found greater liver *Mat1a* expression in offspring from dams that exercised. *Mat1a* encodes methionine adenosyltransferase and functions to convert methionine to AdoMet, which is then used as a methyl donor for several methylation reactions. This suggests that homocysteine is being used for the synthesis of AdoMet (via methionine) resulting in lower serum total homocysteine concentrations in the offspring from dams that exercised. However, in all of the offspring, regardless of maternal diet or exercise, serum total homocysteine concentrations were below levels typically associated with endothelial dysfunction in mice (Dayal & Lentz, 2008). Therefore, it is unlikely that the differences in homocysteine observed in the mice contributed to the beneficial effect of maternal exercise on endothelial function in offspring from obese dams.

Interestingly, despite observing a positive effect of maternal exercise on offspring vascular endothelial function, we found no benefits of exercise on vascular function in the dams. Others have reported that voluntary wheel running for 6 weeks prior to and during pregnancy improved endothelial‐dependent vasodilatation in the mesenteric arteries from dams at gestational day 19 compared to those that were sedentary (Gilbert, Banek, Bauer, Gingery, & Dreyer, 2012). We examined vascular function in the thoracic aorta from dams at a later time point, 2–3 weeks after the pups were weaned. It is plausible that exercise‐related improvements in vascular function are transient and were present during pregnancy in our study but dissipated over time. Futhermore, the effects of exercise on vascular function during pregnancy may be vessel‐specific. Enhanced endothelial‐dependent vasodilatation of mesenteric arteries, but not in aorta, from pregnant mice at gestational day 17–18 compared compared to nonpregnant mice (Cooke & Davidge, 2003) was reported suggesting mesenteric arteries are more susceptable to alterations in vascular function than aorta during pregnancy. Additional research is needed to determine the effect of exercise during pregnancy on vascular function of different vessels in the dams.

The early postnatal/suckling period is also an important time point that has long‐term impacts on vascular function. For example, pups from control dams that were cross‐fostered to high‐fat diet‐fed dams during the suckling period had lower endothelial‐dependent vasodilatation compared to pups from control diet‐fed dams that were not cross‐fostered (Khan et al., 2005). Further research on the impact of early suckling diet on offspring vascular function is required.

While this study provides evidence of a beneficial effect of maternal exercise on endothelial function in offspring from obese dams and insight into potential pathways underlying this observation, we acknowledged that there are some caveats to this study. One of which is that the running wheels were in the cages at the time of breeding, thus, the male mice had access to the running wheels for ~24 hr. We chose this approach to avoid stress associated with forced exercise protocols in the dams. Recent studies have reported that paternal exercise (>3 weeks) can mitigate the detrimental effects of paternal obesity on offspring glucose tolerance, adiposity, and pancreatic β‐cell mass; vascular function was not assessed (McPherson, Lane, Sandeman, Owens, & Fullston, 2017; McPherson, Owens, Fullston, & Lane, 2015; Stanford et al., 2018). In our study, the mating pairs were set up nightly and males were removed immediately after breeding was confirmed (visible by a vaginal plug). Thus, the effect of paternal exercise in our study is likely negligible. However, more research is warranted to determine the amount of maternal and paternal exercise needed to improve offspring vascular health. Another caveat to our study is that the offspring from the exercised obese dams may have had higher levels of physical activity. A recent study reported that adult offspring from dams that exercised during pregnancy had higher levels of physical activity (Eclarinal et al., 2016).

In summary, our data suggest that maternal exercise improves vascular endothelial function in offspring from obese dams, but that these effects are dependent on the offspring's diet. The improvement in endothelial function was only observed in offspring fed the western diet. This study also offers insight into the potential role of one‐carbon metabolism in the programming of endothelial function by maternal exercise. Overall, our findings suggest that exercise during pregnancy may be an effective nonpharmacological intervention to improve vascular health in offspring from mothers with gestational obesity.

## CONFLICT OF INTEREST

None.

## AUTHOR CONTRIBUTION

AMD and IL designed the research. NPB, SG, DCT, and REA conducted the experiments. PB provided expertise on the endothelial cell functionality experiments. JWM provided expertise and analyses of one‐carbon nutrient metabolites. NPB wrote the first draft of the manuscript. All authors contributed to writing the manuscript and approved the final version of the manuscript.

## ETHICAL STATEMENT

All procedures were performed according to the guidelines and with the approval of the University of British Columbia Animal Care Committee.
